# Pre-contrast ShMOLLI T1 mapping in cardiac AL amyloidosis

**DOI:** 10.1186/1532-429X-14-S1-O76

**Published:** 2012-02-01

**Authors:** Theodoros Karamitsos, Sanjay M Banypersad, Daniel Sado, Viviana Maestrini, Vanessa Ferreira, Stefan K Piechnik, Matthew D Robson, Philip N Hawkins, Stefan Neubauer, James Moon

**Affiliations:** 1 MRI, The Heart Hospital, London, UK; 2 Dept. of Cardiovascular Medicine, University of Oxford Centre for Clinical Magnetic Resonance Research (OCMR), Oxford, UK; 3 National Amyloidosis Centre, Royal Free Hospital, London, UK

## Background

Multi-organ disease with cardiac involvement carries a very poor prognosis in Systemic AL Amyloidosis. The risk of nephrogenic systemic fibrosis is a significant obstacle in assessing cardiac status using CMR in patients with systemic AL amyloidosis who have advanced renal failure. Measurement of myocardial T1 values has been limited until now, due to long breath-hold times. We have developed a robust and clinically applicable technique for diagnosing cardiac amyloidosis by measuring absolute myocardial T1 values using the single breath hold, Shortened Modified Look-Locker Inversion Recovery (ShMOLLI) sequence without gadolinium administration.

## Methods

Thirty-five patients (23 males, 12 females, mean age 60 years) with systemic AL amyloidosis underwent conventional CMR scanning with cine imaging and late gadolinium imaging as well as ShMOLLI pre-contrast T1-mapping between both centres; all patients had an eGFR of >30ml/min. Myocardial T1 values from the basal septum in the apical 4-chamber view were measured and compared against cardiac biomarkers, and ECG data. Results were compared to normal controls (n=54). Conventional clinical assessment using the Mayo staging system ranked cardiac involvement as definite, probable and none.

## Results

**Figure 1:**
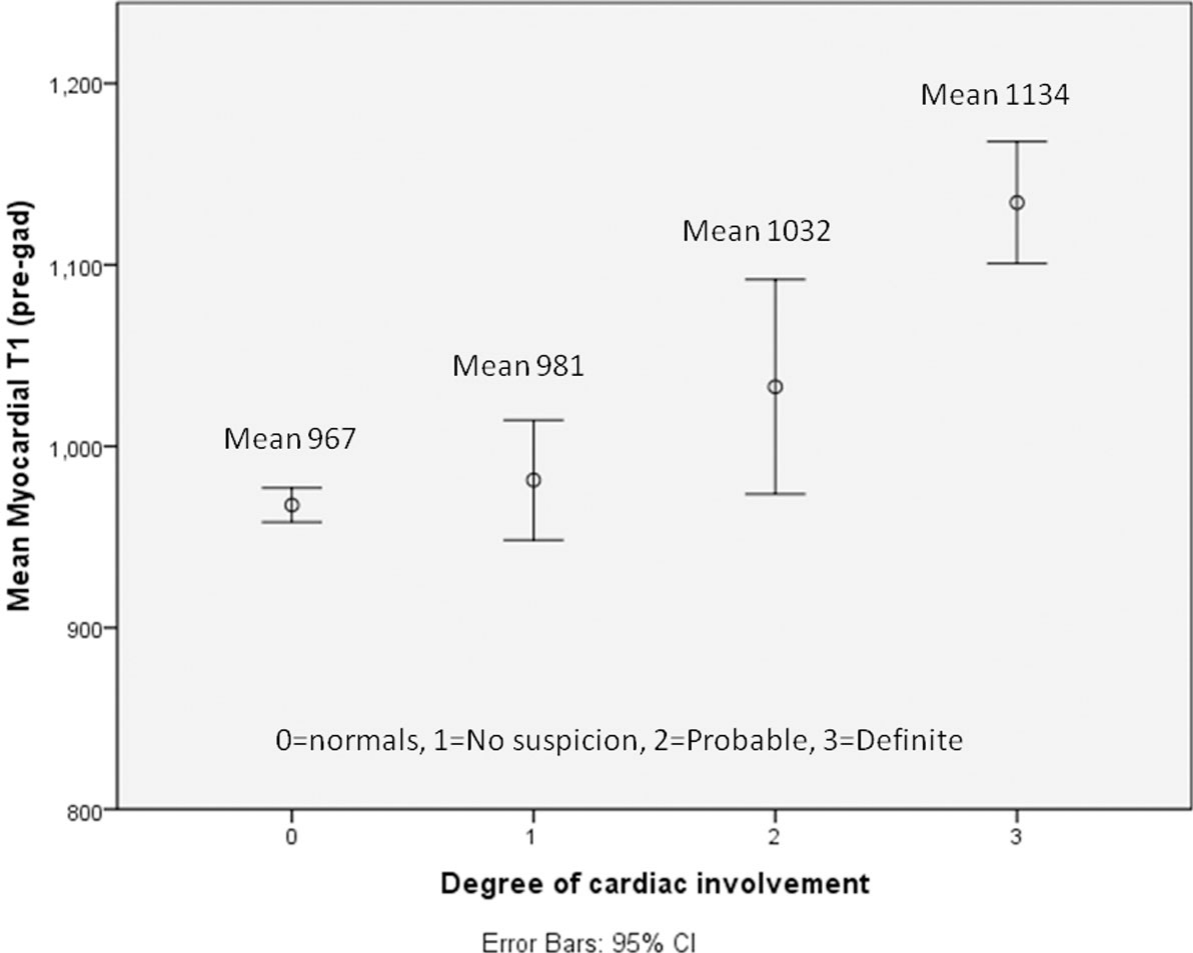
showing pre-contrast, mean myocardial T1 in patients with cardiac AL amyloidosis, subgrouped into pre-test clinical probability of cardiac involvement.

**Figure 2:**
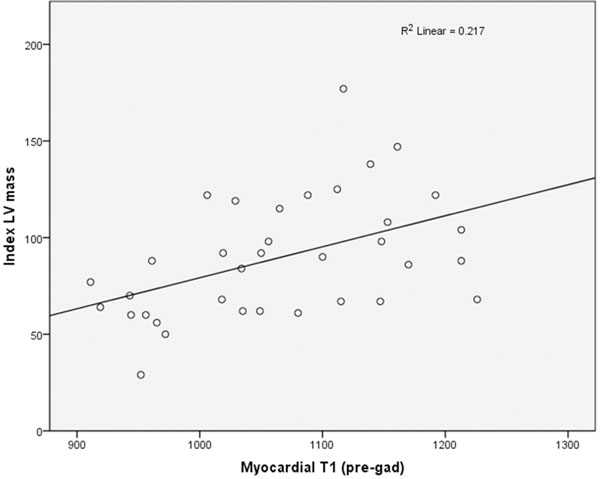
showing the correlation between pre-contrast, mean myocardial T1 by ShMOLLI and mean indexed LV mass.

Myocardial T1 was significantly higher in all patients with systemic AL amyloidosis compared to normals (1057 vs 967, P<0.001). When assessed against pre-test probability of cardiac involvement based on clinical evaluation, myocardial T1 in patients with probable and definite cardiac disease was significantly higher than normals (P<0.005) (see figure 1). Myocardial T1 correlated linearly with indexed LV mass (R^2^ 0.21, P<0.005), BNP (R^2^ 0.49, P<0.005) and inversely with ejection fraction (R^2^ 0.31, P<0.005) and QRS voltage on ECG (R^2^ 0.32, P<0.005) (see figure 2). Correlations were not observed with markers of functional assessment such as NYHA class and ECOG status, possibly due to confounding variables such as co-existing peripheral neuropathy and degree of fluid overload.

## Conclusions

Using the ShMOLLI sequence to measure absolute myocardial T1 times in patients with systemic AL amyloidosis is a safe and accurate method for determining the presence or otherwise of cardiac involvement by amyloid and it correlates well with currently accepted measures of cardiac dysfunction in amyloidosis.

## Funding

GSK, SKP, VMF, MDR funded by the NIHR Oxford Biomedical Research Centre Programme.

